# Hyperuricemia and chronic kidney disease: to treat or not to treat

**DOI:** 10.1590/2175-8239-JBN-2020-U002

**Published:** 2021-03-05

**Authors:** Federica Piani, Fumihiko Sasai, Petter Bjornstad, Claudio Borghi, Ashio Yoshimura, Laura G. Sanchez-Lozada, Carlos Roncal-Jimenez, Gabriela E. Garcia, Ana Andres Hernando, Gabriel Cara Fuentes, Bernardo Rodriguez-Iturbe, Miguel A Lanaspa, Richard J Johnson

**Affiliations:** 1 University of Colorado School of Medicine, Division of Renal Diseases and Hypertension, Department of Medicine, Aurora, CO, USA.; 2 University of Bologna, Department of Medical and Surgical Sciences, Bologna, Italy.; 3 Shin-Yokohama Daiichi Hospital, Yokohama, Kanagawa, Japan.; 4 Hospital Universitario de Maracaibo, Instituto de Investigaciones Científicas, Ivic-Zulia, Maracaibo, Venezuela.; 5 Rocky Mountain VA Medical Center, Aurora, CO, USA.

**Keywords:** Hyperuricemia, Uric Acid, Acute Kidney Injury, Renal Insufficiency, Chronic, Allopurinol, Cardiovascular Disease, Hiperuricemia, Ácido Úrico, Lesão Renal Aguda, Insuficiência Renal Crônica, Alopurinol, Doenças Cardiovasculares

## Abstract

Hyperuricemia is common in chronic kidney disease (CKD) and may be present in 50% of patients presenting for dialysis. Hyperuricemia can be secondary to impaired glomerular filtration rate (GFR) that occurs in CKD. However, hyperuricemia can also precede the development of kidney disease and predict incident CKD. Experimental studies of hyperuricemic models have found that both soluble and crystalline uric acid can cause significant kidney damage, characterized by ischemia, tubulointerstitial fibrosis, and inflammation. However, most Mendelian randomization studies failed to demonstrate a causal relationship between uric acid and CKD, and clinical trials have had variable results. Here we suggest potential explanations for the negative clinical and genetic findings, including the role of crystalline uric acid, intracellular uric acid, and xanthine oxidase activity in uric acid-mediated kidney injury. We propose future clinical trials as well as an algorithm for treatment of hyperuricemia in patients with CKD.

## Resumo

A hiperuricemia é comum na doença renal crônica (DRC) e pode estar presente em até 50% dos pacientes que se apresentam para diálise. A hiperuricemia pode ser secundária ao comprometimento da taxa de filtração glomerular (TFG) que ocorre na DRC. No entanto, ela também pode preceder o desenvolvimento da doença renal e mesmo prever uma DRC incidente. Estudos experimentais de modelos hiperuricêmicos descobriram que tanto o ácido úrico solúvel quanto o cristalino podem causar danos renais significativos, caracterizados por isquemia, fibrose tubulointersticial e inflamação. Entretanto, a maioria dos estudos de randomização Mendeliana falhou em demonstrar uma relação causal entre o ácido úrico e a DRC, e os ensaios clínicos têm apresentado resultados variáveis. Aqui sugerimos explicações potenciais para os achados clínicos e genéticos negativos, incluindo o papel do ácido úrico cristalino, do ácido úrico intracelular e da atividade da xantina oxidase na lesão renal mediada por ácido úrico. Propomos ensaios clínicos futuros, bem como um algoritmo para o tratamento de hiperuricemia em pacientes com DRC.

## Introduction

The prevalence of chronic kidney disease (CKD) and hyperuricemia is increasing worldwide^1^. CKD is commonly associated with gout, and the association dates back to the mid-nineteenth century^2^^,^ 3. Numerous epidemiological studies have consistently shown that hyperuricemia independently predicts new onset CKD^4^^-^ 6. In addition, hyperuricemia frequently associates with other risk factors for CKD, such as hypertension and metabolic syndrome^1^. However, most Mendelian randomization studies have failed to find a causal relationship^7^. Clinical trials have also provided inconsistent data; while earlier trials have generally shown benefit, two recent clinical trials found no effects of lowering serum uric acid in participants with type 1 diabetes with CKD or in patients with non-diabetic CKD (Table 1). Furthermore, it remains debated whether asymptomatic hyperuricemia in the absence of gout confers similar risk for CKD as those with gout^4^. Here we discuss the controversy of the role of hyperuricemia in CKD, and critically appraise the therapeutic role of lowering serum uric acid in patients with CKD.

**Table 1:** Clinical studies on uric acid lowering drugs in patients with CKD

Study	n	Study population	Drug	FU	Result
Siu 2006^67^	54	Patients with hyperuricemia and CKD	Allopurinol	12m	Allopurinol helps preserve kidney function during 12 months of therapy compared with controls
Goicoechea 2010^68^	113	Patients with CKD	Allopurinol vs Controls	24m	Allopurinol decreased C-reactive protein and delayed the progression of renal impairment in patients with chronic kidney disease
Hosoya 2014^69^	123	Patients aged 20–75 years, with hyperuricemia and CKD stages 2-3	Topiroxostat vs Placebo	5.5m	Changes in eGFR were not significantly different between topiroxostat and placebo groups
Sircar 2015^70^	93	Patients with CKD stages 3-4	Febuxostat vs Placebo	6m	Febuxostat significantly decrease the decline in eGFR compared to placebo
Xuemei Liu 2018^71^	832	Meta-analysis: 12 RCTs	Allopurinol or Febuxostat	4-24m	The risk of worsening of kidney function or ESRD or death was significantly decreased in the treatment group compared to the control group
Kimura 2018^72^	443	Japanese patients with stage 3 CKD and asymptomatic hyperuricemia	Febuxostat vs placebo	27m	Febuxostat did not mitigate the decline in kidney function
Lee 2019^73^	141	Patients with hyperuricemia and CKD stage 3	Febuxostat vs Allopurinol	5y	Febuxostat reduced serum uric acid level and delayed CKD progression more effectively than allopurinol
Badve 2020^60^	363	Patients with stage 3-4 CKD and no history of gout who had a urinary albumin:creatinine ratio≥265 or an eGFR decrease of at least 3.0 mL/min/1.73 m2 in the preceding year	Allopurinol vs Placebo	26m	Allopurinol did not significantly slow the decline in eGFR compared with placebo
Doria 2020^59^	530	Patients with type 1 diabetes, SUA>4.5mg/dL, and eGFR40~99mL/min/1.73 m2	Allopurinol vs Placebo	38m	No significant differences in CKD progression between allopurinol and placebo were observed
Hsu 2020^74^	6057	Patients with stage 5 CKD prescribed either febuxostat or allopurinol	Febuxostat vs Allopurinol	4y	Febuxostat decreased the rate of progression to dialysis
Sezai 2020^75^	55	Patients with CKD stage 3-4	Febuxostat vs Topiroxostat	1y	Febuxostat had stronger renoprotective and antioxidant effects than topiroxostat

List of abbreviations: CKD, chronic kidney disease; eGFR, estimated glomerular filtration rate; FU, follow-up.

### Pathogenesis of hyperuricemia in ckd

Although uric acid concentrations are tightly regulated in most species, humans lost this regulatory capacity due to a mutational loss in uricase that degrades uric acid to 5-hydroxyisourate and subsequently allantoin^8^. A consequence is that serum uric acid can be increased by dietary intake of purine rich foods, fructose, and alcohol. In turn, regulation of uric acid concentrations is primarily via excretion by the kidney (two-thirds of total elimination) and gut (one-third)^9^. In addition, a small amount of uric acid is metabolized by oxidants to allantoin, triuret or 6-aminouracil^10^. In the kidney, uric acid is freely filtered, with a net 90% reabsorbed in the proximal tubule by different transporters (e.g. urate transporter 1 (URAT1), and organic anion transporter 4 (OAT4)), and with approximately 10% excreted^11^. As kidney function declines, uric acid is retained^12^. However, it has been demonstrated that renal impairment is accompanied by a significant compensatory increase in the fractional excretion of uric acid (FeUA) and in the excretion of uric acid per volume of glomerular filtration^12^. Furthermore, extra-renal uric acid excretion also increases as a compensatory response to reduced kidney excretion of uric acid^13^. Thus, while impaired kidney function will increase serum uric acid levels, its contribution is less than the effects of impaired kidney function on, for example, blood urea nitrogen and creatinine.

While hyperuricemia can result from impaired kidney function, numerous studies have reported that hyperuricemia commonly precedes the development of CKD^14^^-^ 16. This may occur in part because conditions such as obesity, metabolic syndrome, and hypertension are risk factors for CKD and are also commonly associated with hyperuricemia. Proposed mechanisms for the hyperuricemia in these conditions include insulin-dependent reduction in FeUA, hypercholesterolemia-mediated increase in xanthine oxidase (XO) activity, and hypertension-induced afferent arteriolar vasoconstriction with renal retention of uric acid^17^^-^ 19.

A genetic predisposition to hyperuricemia and kidney injury has also been shown. In fact, genetic polymorphisms in the regulation of serum uric acid levels have been associated with estimated glomerular filtration rate (eGFR)^20^. Additionally, some medications commonly administered to patients with CKD, such as diuretics and immunosuppressants, may also raise serum uric acid concentrations^21^.

### Epidemiology of the association

Hyperuricemia is a strong independent risk factor for incident CKD^(14-16, 22-28)^. The relationship of serum uric acid with incident CKD is not linear, but risk shows a rapid increase as serum uric acid concentrations reach 7 mg/dL or more^29^^,^ 30. In contrast, once patients develop CKD, serum uric acid is more variable, with some studies suggesting it is an independent predictor for worsening of CKD^16^^,^ 31 ^,^ 32 whereas others studies suggest it is not^6^^,^ 33. There are also some studies from Japan that suggest a low uric acid may magnify risk for CKD, but this may be due the relatively higher frequency of mutations in the transporter URAT-1 that is associated with severe uricosuria and recurrent acute kidney injury (AKI)^34^^,^ 35.

Some genetic studies also suggest that hyperuricemia may confer risk for CKD, especially in Mexican American, Native American, and Italian populations^20^^,^ 36 ^,^ 37. However, a recent large Mendelian randomization study did not find any association between serum uric acid, eGFR, and CKD^7^.

These controversial results could be a consequence of selection biases due to the heterogeneity of the hyperuricemic population. For example, it may make a difference whether the hyperuricemia is primary (e.g., dietary or from increased synthesis) or secondary (e.g., due to passive retention from attenuated renal excretion due to an impaired eGFR). Another variable could be the level of hyperuricemia, for, as mentioned, the relationship between serum uric acid levels and the development of CKD is not linear but increases exponentially for values of serum uric acid > 7 mg/dL and especially > 9 mg/dL^16^^,^ 29 ^,^ 32 ^,^ 38. It is also plausible that it may relate to whether there is crystal deposition in the kidney, which might be expected to be higher in patients with gout, although people with asymptomatic hyperuricemia may also have silent crystal deposition in joints and other organs^39^^,^ 40. Indeed, gout has been associated with a higher risk of advanced CKD compared to asymptomatic hyperuricemia^41^^,^ 42. It is thus evident how prior studies are not easily generalizable, as distinct subgroups of people with hyperuricemia may show a different risk of CKD.

There is also some evidence that the biologic effects of uric acid to cause kidney disease may be mediated more by the intracellular effects of uric acid as opposed to serum uric acid^43^^,^ 44. Intracellular levels might be higher in the setting where synthesis is stimulated, such as may be observed with high xanthine oxidoreductase (XO) activity. Plasma XO activity is associated with CKD progression and cardiovascular outcomes, independently of serum uric acid^45^^,^ 46. This could potentially explain why serum uric acid may not predict CKD by Mendelian randomization studies, as the polymorphisms in urate transporters that predict hyperuricemia may have different effects on intracellular uric acid concentrations^47^.

### Does uric acid cause kidney injury?

**Figure 1:**
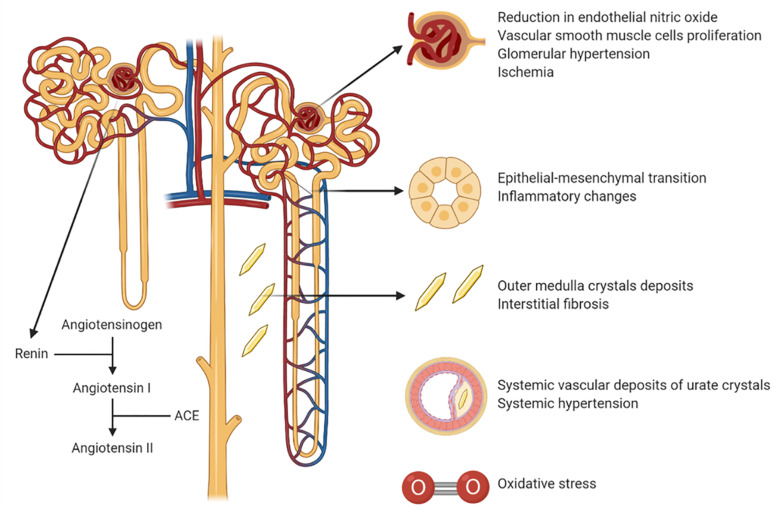
Mechanisms of uric acid-induced kidney injury.

Hyperuricemia is thought to cause kidney injury by both crystal-dependent and crystal-independent mechanisms^48^ (Figure 1).

The crystal-dependent pathway involves the deposition of monosodium urate crystals in the tubules or interstitium in the kidney in the outer medulla that leads to chronic inflammation and tubular damage^48^. Recently it has been suggested that this can be diagnosed by renal ultrasound showing a “hyperechoic” outer medulla, and that it may be present in one-third of patients with gout where it correlates with kidney function^49^. Interestingly, the presence of this microcrystalline nephropathy was not associated with urinary evidence for urate crystalluria, underlying the independence of the two pathophysiologic mechanisms^49^. Of note, urate crystals have also been discovered to deposit in both the aorta and coronary arteries, where they may have a role in plaque formation and vascular calcification^40^. As such, the crystal-dependent pathway may also be a mechanism by which uric acid may be involved in the pathogenesis of atherosclerosis and heart disease.

Soluble, intracellular uric acid may also cause CKD via a crystal-independent process. This may occur by either uptake of uric acid from the circulation or by endogenous generation such as from dietary fructose^50^. The mechanism involves elevations in both systemic and intraglomerular pressure coupled with afferent arteriolar vasoconstriction with impaired renal blood flow that is mediated by activation of the renin angiotensin aldosterone (RAAS) system, a reduction in endothelial nitric oxide bioavailability and the induction of oxidative stress^51^^,^ 52. There is also vascular smooth muscle cell proliferation that leads to an arteriolopathy that impairs autoregulation, and also effects on tubules that include epithelial-mesenchymal changes and inflammatory changes^53^^-^ 55. Indeed, ischemia is one of the main pathology findings in both human and animal kidney of subjects affected by hyperuricemia and gout^56^. Of note, most of the animal studies on hyperuricemia have used the oxonic acid-induced hyperuricemic rat model^57^.

### Clinical trials of uric acid in chronic kidney disease

Experimental trials of uric acid lowering drugs in CKD have been mixed (Table 1). One analysis suggested that a primary reason for the mixed results was that some trials were too short or underpowered to show meaningful progression in the control groups, thus making it difficult to show a benefit in the treatment group. In essence, if the control group does not demonstrate worsening of the underlying disease, it is challenging for any treatment to demonstrate protection. Indeed, studies showing meaningful progression (defined as ≥4 mL/min/1.73m^2^ decrease in the control group over the time course of the study) were associated with a benefit of urate-lowering therapy. This analysis argued for urate-lowering therapy in participants with hyperuricemia and CKD^58^.

More recently, two large clinical trials, the Preventing Early Renal Loss in Diabetes (PERL) and the Controlled Trial of Slowing of Kidney Disease Progression from the Inhibition of Xanthine Oxidase (CKD-FIX) studies, were published in which significant progression did occur in the control groups but for which no benefit in treatment of uric acid levels were noted^59^^,^ 60. The PERL was well designed but the participants who had type 1 diabetes did not have gout, and the majority had normal serum uric acid concentrations. The CKD-FIX also did not enroll participants with gout and included subjects irrespective of their serum uric acid concentration. Both groups also had dropout rates greater than 15 percent which were included in the analysis as they were intention-to-treat studies. Hence, neither study targeted the population at risk, that being participants with hyperuricemia with or without gout, and thus conclusions on treating hyperuricemia in CKD are still unclear.

### Treatment recommendations

Before any recommendations are provided, it is important to discuss the potential toxicities of the various treatments. Allopurinol is a xanthine oxidase inhibitor that is usually well tolerated, but it can be associated with a severe hypersensitivity syndrome mimicking a Stevens Johnson syndrome in individuals carrying the HLA B58 allele^61^. This is especially common in the Asian population. The other common xanthine oxidase inhibitor, febuxostat, does not appear to have this side effect but was associated with increased all-cause and cardiovascular mortality compared to allopurinol in the CARES trial (62), although another recently published trial did not observe any difference between allopurinol and febuxostat on cardiovascular outcomes^63^. In the CARES trial, most of the cardiovascular events occurred after the febuxostat was stopped^64^. Stopping xanthine oxidase inhibitors has been associated with worsening of kidney function in patients with CKD, but only in those who are not on RAAS blockers^65^. Since treatment of xanthine oxidase inhibitors is known to block the RAAS^66^, it is possible that stopping xanthine oxidase inhibitors could cause a rebound activation of the RAAS.

Other uric acid lowering agents include uricosurics, but these are not recommended in patients with CKD as acute rises in urine uric acid may cause transient worsening of kidney function. However, this may be mitigated by combining a uricosuric with a xanthine oxidase inhibitor. Finally, uric acid can also be lowered by recombinant uricases such as pegloticase and rasburicase. However, some individuals may develop antibodies to these agents that can limit their eventual effectiveness.

Clearly, more clinical trials are needed. However, based on the fact that marked hyperuricemia appears to carry significant risk for kidney disease progression that could involve both crystal-dependent and -independent mechanisms, we suggest that treatment should be considered for individuals with serum uric acid concentrations of 8 mg/dL or higher and evidence of progression of their kidney disease, as well as patients with a history of gout irrespective of their underlying serum uric acid concentration. In Table 1 we summarized the main clinical studies on uric acid lowering drugs in patients with CKD. We would recommend assessing if patients are on a RAAS inhibitor before initiating allopurinol (beginning with low doses of 50 mg daily with slow titling to a maximum of 300 mg/daily). All patients are told to stop allopurinol if they develop a rash, and those of Asian ancestry should consider HLA typing prior to drug initiation. Alternatives to allopurinol could include febuxostat or combination uricosuric-xanthine oxidase combinations. Pegloticase can also be used for those with severe and refractory gout.

In summary, hyperuricemia is a risk factor for CKD, and there is strong evidence that it can cause kidney disease by either crystal-dependent or -independent mechanisms. Nevertheless, trials examining the effects of uric acid lowering interventions in CKD have been inconsistent, although this be ascribed to the trials not targeting the participants most likely to benefit, which are those with hyperuricemia and/or a history of gout. Until further trials are done, we still would recommend urate-lowering drugs for this target population provided they show evidence of progression of their kidney disease. Careful attention to the potential toxicities of urate-lowering drugs is needed.
